# Synthetic mesh versus biological mesh to prevent incisional hernia after loop-ileostomy closure: a randomized feasibility trial

**DOI:** 10.1186/s12893-023-01961-4

**Published:** 2023-03-27

**Authors:** Elisa J Mäkäräinen, Heikki T Wiik, Jyrki AO Kössi, Tarja M Pinta, Leena-Mari J Mäntymäki, Anne K Mattila, Matti VJ Kairaluoma, Pasi P Ohtonen, Tero T Rautio

**Affiliations:** 1 Medical Research Center Oulu, Oulu University Hospital, PL 10, Oulu, 90029 Finland; 2 Päijät-Häme Central Hospital, Keskussairaalankatu 7, Lahti, 15850 Finland; 3 Seinäjoki Central Hospital, Hanneksenrinne 7, Seinäjoki, 60220 Finland; 4 Tampere University Hospital, Elämänaukio 2, Tampere, 33520 Finland; 5 Keski-Suomi Central Hospital, Hoitajantie 3, Jyväskylä, 40620 Finland

**Keywords:** Incisional hernia prevention, Loop-ileostomy closure, Synthetic mesh, Biological mesh, Rectal cancer

## Abstract

**Background:**

Incisional hernia is a frequent complication after loop-ileostomy closure, rationalizing hernia prevention. Biological meshes have been widely used in contaminated surgical sites instead of synthetic meshes in fear of mesh related complications. However, previous studies on meshes does not support this practice. The aim of Preloop trial was to study the safety and efficacy of synthetic mesh compared to a biological mesh in incisional hernia prevention after loop-ileostomy closure.

**Methods:**

The Preloop randomized, feasibility trial was conducted from April 2018 until November 2021 in four hospitals in Finland. The trial enrolled 102 patients with temporary loop-ileostomy after anterior resection for rectal cancer. The study patients were randomized 1:1 to receive either a light-weight synthetic polypropylene mesh (Parietene Macro™, Medtronic) (SM) or a biological mesh (Permacol™, Medtronic) (BM) to the retrorectus space at ileostomy closure. The primary end points were rate of surgical site infections (SSI) at 30-day follow-up and incisional hernia rate during 10 months’ follow-up period.

**Results:**

Of 102 patients randomized, 97 received the intended allocation. At 30-day follow-up, 94 (97%) patients were evaluated. In the SM group, 1/46 (2%) had SSI. Uneventful recovery was reported in 38/46 (86%) in SM group. In the BM group, 2/48 (4%) had SSI (p > 0.90) and in 43/48 (90%) uneventful recovery was reported. The mesh was removed from one patient in both groups (p > 0.90).

**Conclusions:**

Both a synthetic mesh and biological mesh were safe in terms of SSI after loop-ileostomy closure. Hernia prevention efficacy will be published after the study patients have completed the 10 months’ follow-up.

## Introduction

Loop-ileostomy closure leads to incisional hernia (IH) in one fifth to one third of patients [1–3] and up to 20–40% of patients may require further repair [4–6]. However, IH rate may be underreported [4, 7]. The risk factors for IH include high body mass index and American Society of Anesthesiologists (ASA) class 3–4 [2, 8].

The synthetic mesh does not increase the surgical site occurrence rate compared to biological mesh in large randomized controlled trial (RCT) including clean and clean contamined ventral hernia repair [9]. IH prevention with a type of medical mesh at temporary stoma closure has thus far been a rarely explored topic. Liu et al. published an IH rate of 6% with a synthetic polypropylene mesh versus 36% in controls without mesh in a retrospective case-control study [1]. Likewise, Warren et al. published hernia rates of 1% with a synthetic mesh (SM) vs. 17% without mesh in a retrospective cohort study after different types of stoma closures [10]. Maggiori et al. used a biological mesh (BM) in retrorectus position in a retrospective case-control setting with 3% hernia rate in the BM group versus 19% in controls [11]. The largest randomized controlled trial to date compared intra-abdominal BM to a control group without a preventive mesh applied at the surgical site [3]. The use of BM resulted in a decreased IH rate compared to the non-mesh group (12% vs. 20%, respectively). Shaw et all published a prospective case serie using synthetic prophylactic mesh in rectorectus space for 20 patients. No surgical site occurrence was reported within the mean follow up time of 20 months [12]. In a recent systematic review, the use of prophylactic mesh was considered safe in terms of SSI, seroma and anastomotic leakage [13]. Additionally, mesh decreased the hernia rate compared to non-mesh closure.

Biological meshes have been considered safer than synthetic ones for use in contaminated surgical sites. However, clinical evidence does not support the practice [9, 14]. Additionally, synthetic meshes may be more efficient in hernia prevention and they cost less [9]. To our knowledge, the results of randomized clinical trials comparing synthetic and biologic meshes in IH prevention after temporary stoma closure have not been published previously.

The objective of the Preloop trial is to compare the safety and efficacy of SM (Parietene Macro™, Medtronic, Minneapolis, MN, USA) and BM (Permacol™, Medtronic, Minneapolis, MN, USA) in a non-inferiority setting after temporary loop ileostomy closure during both short- and long-term follow-up. In this paper, we report the 30-day SSIs as primary outcomes of the study. The IH rate will be reported after the study patients reach the 12-month follow-up.

## Materials and methods

### Study design

The Preloop trial was designed as a randomized, controlled, multi-center feasibility study comparing the safety and efficacy of SM to BM in retrorectus space as ileostomy site IH prevention after loop-ileostomy closure. The protocol has been published previously [15]. The trial was registered at Clinical Trials (NCT03445936, 26/02/2018) prior to enrollment of the first patients. The study hypothesis was that the more affordable SM is equally safe and effective in IH prevention after loop-ileostomy closure compared to the more expensive BM. The main endpoints in this trial were SSIs at 30-day follow-up defined by Centers for Disease Control and Prevention definition for SSI and graded with the Clavien-Dindo Classification. The second primary outcome was the incidence of hernias clinically or on CT scan at 10 months after closure of the stoma. The results of the secondary outcome will be later reported. The secondary endpoints are other complications within 30 days of surgery graded with the Clavien-Dindo classification, reoperation rate, operating time, length of stay, quality of life measured with RAND-36, and incidence of hernia over a five-year follow-up period.

### Patients and study setting

**Fig. 1:**
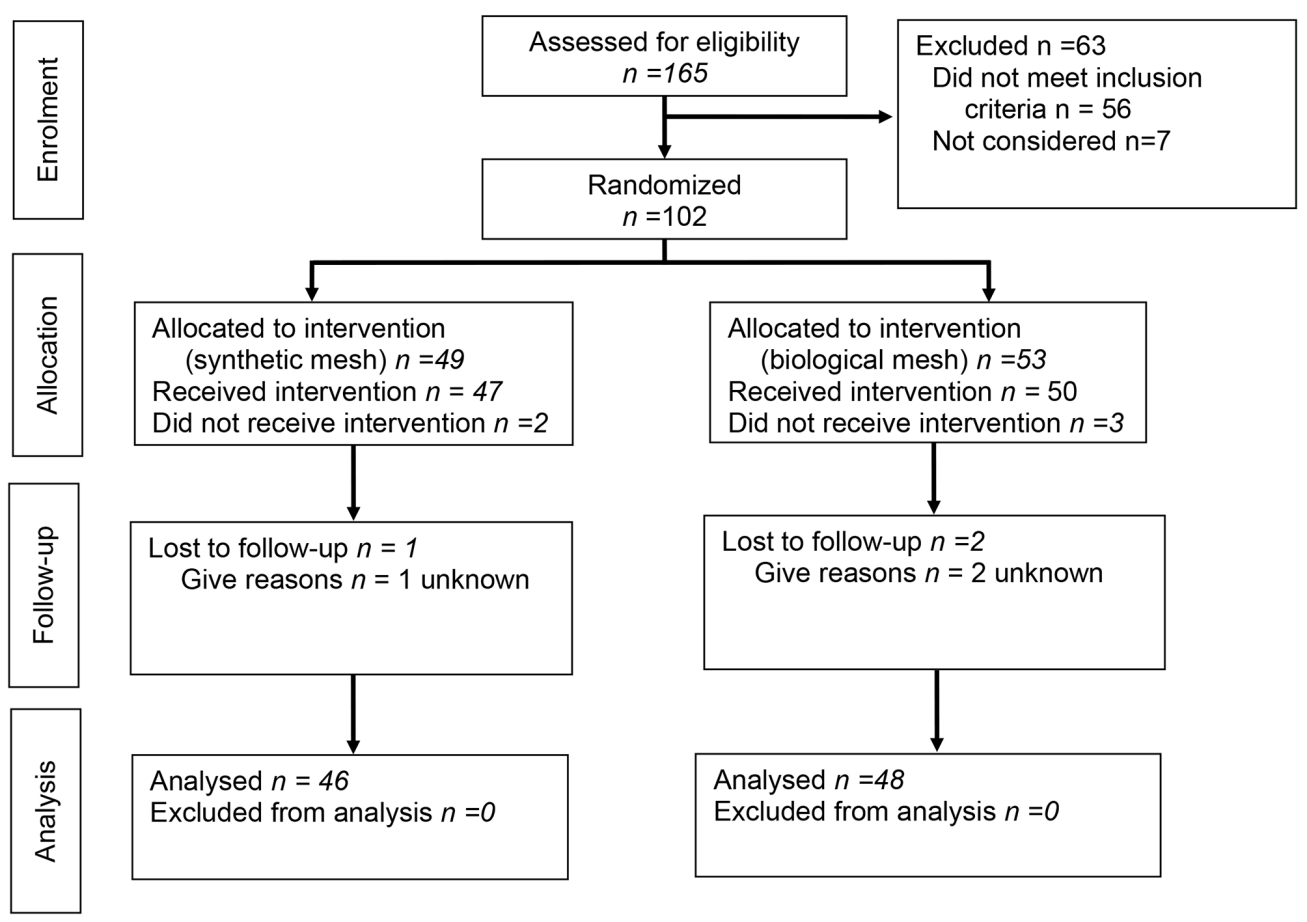
Flow Chart

The study patients were operated on in four Finnish hospitals (Oulu and Tampere University Hospitals, Seinäjoki and Keski-Suomi Central Hospitals). All adult (> 18 years old) patients operated on with curative intent by anterior resection with total mesorectal excision and protective loop-ileostomy for rectal adenocarcinoma, were considered for inclusion (Fig. 1). Exclusion criteria included ASA class IV–V, concurrent or previous other malignant tumors within 5 years, T4b tumors with multiorgan resection, emergency procedures and primary rectal surgery with major concomitant procedures (e.g. hepatectomy, other intestinal resection), metastatic disease and pregnancy. Originally, patients receiving adjuvant chemotherapy were excluded. However, after interim safety analysis showing no adverse patient events, it was decided to include these patients in the study also. The study protocol modification was approved by the Oulu University Hospital Ethics Committee.

### Randomization and blinding

The study was designed as a randomized non-inferior feasibility trial, with 1:1 randomization allocation. Eligible patients were randomized to undergo loop-ileostomy closure with either synthetic light-weight mesh or BM in retrorectus space. Separate randomization lists were generated for each study center. Patients were allocated to study groups according to a computer-generated list, compiled by a biostatistician not involved in the clinical care of trial patients. Randomization was performed in blocks, where block size varied randomly between two, four and six. The randomization group was disclosed in the patient hospital records, and therefore blinding of the personnel and the patient could not be guaranteed during the follow-up.

### Procedure and follow-up

The procedure is described in detail in the protocol publication [15]. Both the SM and BM were applied using identical methods. The re-establishment of intestinal continuity was achieved through the ostomy trephine either by staples or by hand-sewn anastomosis, according to the surgeon’s preference. The posterior rectus sheath was closed with interrupted slowly absorbable monofilament sutures (PDS Plus Antibacterial Suture, Ethicon, Johnson & Johnson, Somerville, NJ). The retrorectus space was bluntly dissected to create the space for the mesh. Four interrupted slowly absorbable monofilament sutures in the corners of the mesh were used to fix the mesh to the underlying posterior rectus sheath. After mesh fixation, the anterior rectus sheath was closed with interrupted slowly absorbable monofilament sutures. The skin defect was closed by a running subcutaneous purse-string suture with a 2.0 polyglactin thread (Coated Vicryl Plus Antibacterial Polyglactin 910 Suture, Ethicon, Johnson & Johnson, Somerville, NJ). The antibiotic prophylaxis was given according to hospital’s protocol before the operation. No antibiotics were continued after the loop-ileostomy closure. Parastomal hernia detected at the closure and defined as intra-abdominal contents protruding through the stomal orifice, was reported. The time to insert the mesh was recorded from the end of closing the posterior rectus sheath to start to closing the anterior rectus sheath.

The size of the BM was 10 cm x 5 cm, which was a compromise between size and cost. The SM was trimmed to fit the width of the retrorectus space. The study patients were interviewed and examined at the 30-day follow-up. An electronic case report form (eCRF) was filled out for the visit and the patient filled out a RAND-36 quality of life questionnaire. The patients were interviewed about their recovery and readmissions. All complications that had occurred during the 30-day follow-up period were recorded. Readmissions and re-operations were recorded. All complications and side-effects were reported on a separate eCRF. Study patient follow-ups occur at 30 days, 10 months, 3 years and 5 years post procedure. All study patients are evaluated at the outpatient clinic at follow-up visits. CT scan is performed at 10 months’ follow-up visit as part of the routine protocol for rectal adenocarcinoma follow-up.

### Outcomes

The primary outcome of the study was SSI rate during the 30-day follow-up, as defined by the Centers for Disease Control and Prevention. The second primary outcome, IH rate, will be published after all the study patients have reached the 12-month follow-up. The secondary outcomes were complications within 30 postoperative days (severity based on Clavien-Dindo Classification), reoperation rate, operative time, length of hospital stay, quality of life and IH rate during the long-term follow-up.

### Statistical analysis and sample size

The synthetic meshes are considered safe at contaminated surgical site in terms of SSI [9, 14]. We estimated that 45 patients per group would give us a reliable estimate for the future sample size calculations of the non-inferiority of synthetic mesh compared to biological mesh [14]. Additionally, we will follow up the patients for the IH rate comparing both meshes. Taking into account a possible 10% drop-out rate, our aim was to have 50 patients per group. As two randomized patients were incorrectly classified as nonrandomized by the software, a total of 102 patients were enrolled.

All analyses were primarily performed according to the intention-to-treat (ITT) principle. In the ITT analyses, the participants were analyzed in a randomized order. Additionally, per-protocol analyses were conducted to safeguard against the risk of falsely claiming non-inferiority. Between-group comparisons of continuous variables were performed by Student’s t-test or Mann-Whitney U-test, the latter if heterogeneous variances persisted. Categorical data was compared using χ^2^ or Fisher’s exact test. Repeatedly measured continuous data were analyzed by linear mixed model (LMM) using individuals as random effects. The covariance pattern for LMM was chosen according to Akaike’s information criteria. Two-tailed p-values are reported. Analyses were performed using SPSS (version 27 or higher) for windows and SAS (version 9.4 or higher).

### Availability of data and materials

The datasets generated and/or analyzed during the current study are not publicly available due to Finnish laws on privacy protection but are available from the corresponding author on reasonable request.

## Results

Between April 2018 and November 2021, 102 patients were enrolled and randomized. A total of 97 patients received the allocated intervention and 94 patients were evaluated at the 30-day follow-up (Fig. 1). The patient characteristics in both randomization groups are presented in Table 1. All operations were classified as contamination class II (clean contaminated). The details of the operations and discharge are presented in Table 2.

**Table 1:** Patient Demographics

	Synthetic mesh (n = 47)	Biological mesh (n = 50)	
Gender			
Female	18 (38)	18 (36)	
Male	29 (62)	32 (64)	
Age	62 ± 9.1	66 ± 11.6	
Body Mass Index	25.5 ± 4.06	25.6 ± 4.7	
ASA class	2.2 ± 0.6	2.2 ± 0.6	
High blood pressure	25 (50)	22 (47)	
Asthma/COPD	3 (6)	2 (4)	
Diabetes	4 (9)	8 (16)	
Immunosuppression	1 (2)	3 (6)	
Current smoker	4 (9)	3 (6)	
Previous hernia of any kind	2 (4)	7 (14)	
Adjuvant therapy	8 (17)	9 (18)	

Nominal variables are reported as counts and percentages (in parentheses); continuous variables are reported as means and standard deviations.

**Table 2:** Operation and Recovery

	Synthetic mesh (n = 47)	Biological mesh (n = 50)	P-value
Time from anterior resection (months)	4.1 ± 2.2	4.4 ± 2.6	0.45
Operating time (min)	79 ± 25.8	76 ± 25.8	0.51
Time to insert the mesh (min)	13.3 ± 7.0	15.1 ± 11.3	0.35
Parastomal hernia detected at stoma closure	5 (11)	8 (16)	0.31
Ileus during the hospital stay	8 (16)	5 (11)	0.42
Discharge straight to home from the hospital	46 (96)	46 (98)	> 0.90
Length of hospital stay (days)	3.2 ± 0.3	3.3 ± 0.3	0.91

Nominal variables are reported as counts and percentages (in parentheses); continuous variables are reported as means and standard deviations.

At the 30-day follow-up, SSI occurred in one patient in the SM group (2%) and two patients in the BM group (4%) (difference − 2, 95% confidence interval − 12 to 8, p > 0.90). In the SM group, one patient had Clavien-Dindo 4 anastomotic leakage with re-operation and mesh removal. In the BM group, one complication was a Clavien-Dindo 2 superficial wound infection treated with oral antibiotics. The other was a Clavien-Dindo 3b abscess requiring re-operation and mesh removal. A total of 38 patients (86%) in the SM group and 43 patients (90%) in the BM group had a completely healed surgical wound by the 30-day follow-up (Table 3). Readmission was required for two patients in the SM group due to rectal bleeding and the anastomotic leakage mentioned above. In the BM group, a patient with SSI was re-admitted after discharge. No differences existed in length of stay, operating time and other complications between the groups. All results are summarized in Table 2. Quality of life and its alterations within recovery as a secondary outcome will be reported in later publications related to the trial.

**Table 3:** Results at 30 days follow-up

	Synthetic mesh (n = 46)	Biological mesh (n = 48)	Difference %	95% CI for the difference	P value
SSI	1 (2)	2 (4)	-2.0	-12.0 to 7.7	> 0.90
Readmission	2 (4)	1 (2)	2.3	-7.1 to 12.6	0.61
Reoperation	1 (2)	1 (2)	0.1	-8.9 to 9.4	> 0.90
Mesh removed	1 (2)	1 (2)	0.1	-8.9 to 9.4	> 0.90
Wound status					
Completely healed	38 (83)	43 (90)	7.0	-7.4 to 21.5	
Wound opened superficially	5 (11)	3 (6)			
Wound opened down to fascial level	2 (4)	2 (4)			
Pain, erythema	1 (2)	0			

Nominal variables are reported as counts and percentages (in parentheses).

## Discussion

The 30-day results of this trial indicate that SMs are safe in contaminated surgical sites and comparable to BMs in IH prevention. The complete wound healing rate was reasonable in both groups considering surgical site contamination.

The advantages of this trial are its randomized multi-center set-up, homogenous patient population and standardized operative techniques. The study is limited by the small number of patients in both groups and the non-blinded manner of the study. The width of the BM was narrower than the width of the SM and the size of the mesh was standardized for all the patients despite the size of the defect. That may lead to biased results in hernia prevention.

There were no differences in the complications, wound healing rate, the length of stay nor the operating time. Therefore, the only difference in the short-term results is mesh price. The longer-term follow-ups will reveal possible differences in IH rate and reoperations related to IH and mesh complications. According to the short-term results of this study, the use of the much more expensive BM at loop-ileostomy closure sites or contaminated surgical sites cannot be justified over the use of SM during a short-term follow-up.

Based on the short-term results of this study, light-weight SM is safe for IH prevention at loop-ileostomy closure sites and its use should be evaluated further. The long-term follow-up will reveal potential cost/benefit relationships among the SM and BM groups in terms of quality of life. The results of this trial should favor the use of SM over BM also at other contaminated surgical sites.

## Data Availability

The datasets generated and/or analyzed during the current study are not publicly available due to Finnish laws on privacy protection but are available from the corresponding author on reasonable request.
